# Evaluation of Clinical Efficacy of 1.2% Rosuvastatin Hydrogel as an Adjunct to Scaling and Root Planing in Generalized Chronic Periodontitis

**DOI:** 10.7759/cureus.61008

**Published:** 2024-05-24

**Authors:** Amit R Pawar, Arvina Rajasekar

**Affiliations:** 1 Periodontics, Saveetha Dental College and Hospitals, Saveetha Institute of Medical and Technical Sciences, Saveetha University, Chennai, IND

**Keywords:** scaling and root planing, local drug delivery, periodontitis, periodontal regeneration, rosuvastatin

## Abstract

Background

Periodontitis, characterized by chronic inflammation and tissue destruction, remains a significant public health concern. Conventional treatment like scaling and root planing (SRP) is effective but often augmented with adjunctive therapies to improve outcomes. Local drug delivery (LDD) systems containing pharmacological agents offer targeted treatment with reduced systemic side effects. Rosuvastatin (RSV), known for its anti-inflammatory and tissue regenerative properties, has shown promise in periodontal therapy. This prospective clinical trial assessed the effectiveness of 1.2% RSV hydrogel as an adjunct to SRP in managing generalized chronic periodontitis.

Methods

Thirty patients were grouped into Group A (SRP alone) and Group B (SRP + 1.2% RSV hydrogel). Clinical measurements, such as the modified sulcular bleeding index (mSBI), probing pocket depth (PPD), and clinical attachment level (CAL), were documented both at the beginning of the study and after three months. Statistical analysis was performed using SPSS software. A p-value of less than 0.05 was considered statistically significant.

Results

Participants in Group B showed significant improvements in mSBI (from 2.34 ± 0.59 to 1.01 ± 0.29), PPD (from 7.36 ± 1.12 mm to 4.63 ± 0.88 mm), and CAL (from 8.56 ± 1.22 mm to 5.90 ± 1.24 mm) compared to Group A at the three-month follow-up. The mean values of these parameters decreased significantly in both groups from baseline to three months. However, the reductions were more substantial in Group B, indicating the beneficial effect of RSV hydrogel adjunctive therapy.

Conclusion

The study demonstrates the efficacy of 1.2% RSV hydrogel employed as a localized drug in enhancing the outcomes of SRP for generalized chronic periodontitis. The adjunctive use of RSV hydrogel led to noteworthy enhancements in clinical parameters, highlighting its potential in periodontal therapy.

## Introduction

Periodontitis is a persistent inflammatory condition affecting the tissues that support the teeth, characterized by the deterioration of the periodontal ligament and alveolar bone [1]. Periodontal pathogenesis involves the formation of dental plaque, predominantly gram-negative organisms. Later because of host-bacterial interaction, the inflammation progresses, resulting in pocket formation, mobility of the tooth, and eventually tooth loss [2,3]. Understanding these processes is vital for effective prevention and intervention in periodontal diseases [4].

Effective management involves addressing the underlying inflammation, often achieved through scaling and root planing (SRP), which aims to remove dental plaque [5,6]. However, recent advancements in periodontal care have explored adjunctive therapies to augment the outcomes of traditional interventions. Systemic antibiotics are often used adjunctively with SRP in cases of generalized chronic periodontitis. They target specific pathogens and control inflammation [7,8]. They are considered mostly in combination with surgical intervention for comprehensive periodontal management. Careful patient selection, microbiological assessment, duration of usage, and periodic reevaluation are crucial to assess their effectiveness while minimizing risks of side effects and resistance [9].

Another promising avenue involves the utilization of local drug delivery (LDD) systems. LDD agents like gels, fibers, microspheres, or chips are designed for targeted treatment of periodontal diseases [10]. They offer advantages such as reduced systemic side effects and enhanced patient compliance. Typically used alongside SRP, these agents, containing antimicrobials or anti-inflammatory drugs, focus on the affected area, providing sustained release for effective periodontal therapy [11]. Ongoing research aims to improve formulations and delivery methods for optimized treatment.

In this context, our study focused on evaluating the effectiveness of 1.2% rosuvastatin (RSV) hydrogel as a potential adjunct to SRP. RSV, a member of the statin group of drugs, traditionally used for systemic cholesterol reduction, has garnered attention for its anti-inflammatory and tissue regenerative properties [12]. Its capacity to reduce inflammation and oxidative stress, enhance angiogenesis, and promote cellular differentiation and proliferation makes it an attractive candidate for periodontal tissue regeneration [13]. This prospective clinical study aimed to assess the efficacy of locally administered 1.2% RSV hydrogel as a supplement to SRP in treating generalized chronic periodontitis.

## Materials and methods

Study population

Before initiating this investigation, the study protocol received approval from the Institutional Review and Ethical Committee, Saveetha Dental College and Hospitals, Chennai, India, with the reference number IHEC/SDC/PERIO-2205/23/307. Informed consent was taken from all patients involved in the research. The sample size was calculated using G Power software, Version 3.1.9.4. The sample size obtained was 30 with a power of 80% and an alpha error of 95% confidence level. This was based on mean and standard deviation values obtained from previous research [14]. Thirty outpatients were recruited from the Department of Periodontology and Implantology at Saveetha Dental College and Hospitals in Chennai. Both male and female patients aged 25 to 55 years, diagnosed with generalized chronic periodontitis and possessing a minimum of 20 natural teeth, were considered eligible for the study. Systemically compromised patients, patients who had taken antibiotics within the previous three months, pregnant or lactating women, and smokers were excluded from the study. Among 30 patients, 15 patients underwent SRP only (Group A - Control), while the remaining 15 patients underwent SRP along with 1.2% RSV hydrogel application (Group B - Test).

Clinical parameters

The clinical data which is composed of modified sulcular bleeding index (mSBI) as outlined by Mombelli in 1987 [15], probing pocket depth (PPD), and clinical attachment level (CAL) were meticulously recorded with UNC-15 probe by using a customized acrylic stent for reference at both baseline and three-month intervals. PPD was recorded from the gingival margin to the base of the sulcus and CAL was recorded from the cementoenamel junction to the base of the sulcus.

Preparation of 1.2% RSV hydrogel

**Figure 1:**
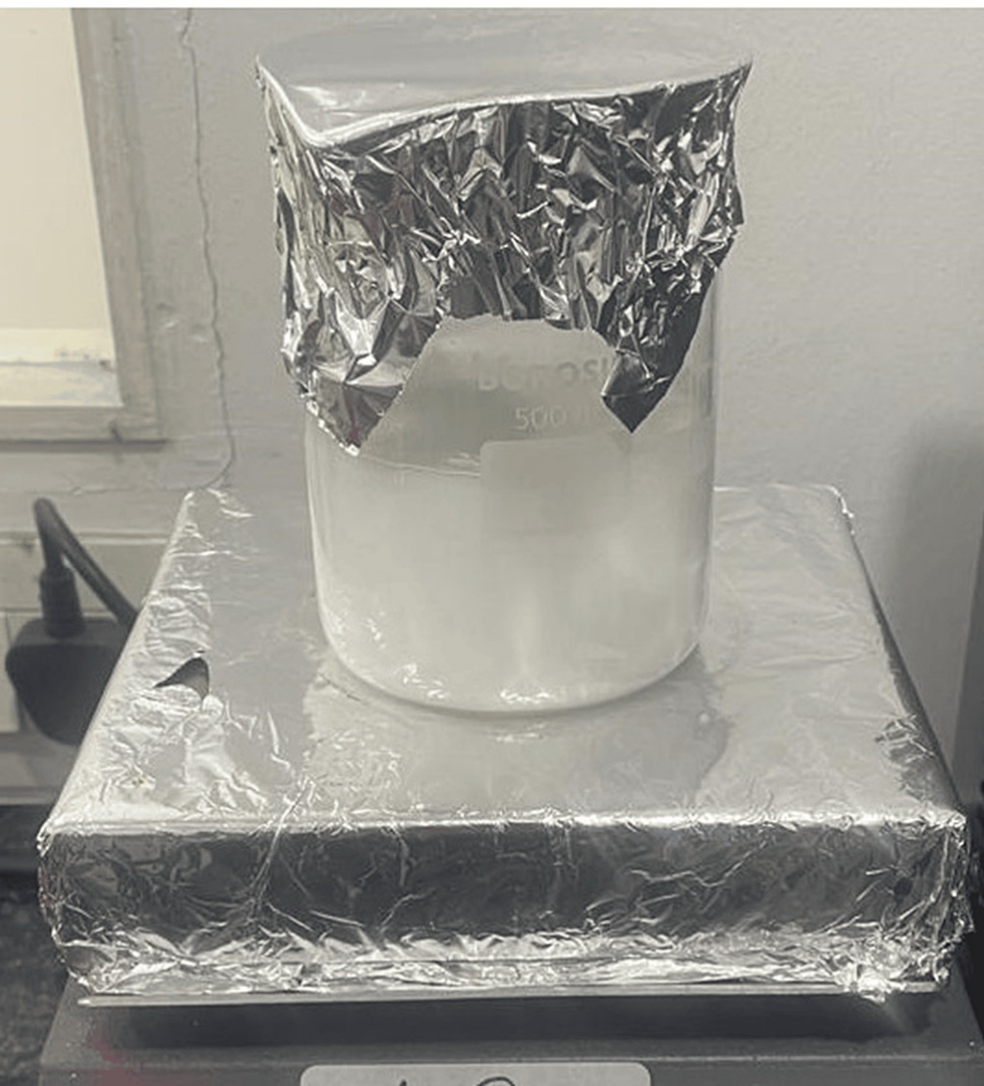
Prepared 1.2% rosuvastatin hydrogel

To prepare a 1.2% RSV hydrogel, five tablets of 10 mg RSV were crushed into powder and dissolved in 4.87 ml of distilled water. Subsequently, 750 microliters of this solution were combined with 3 g of sodium alginate, serving as a gelling agent. The volume was adjusted using distilled water as necessary. The mixture underwent thorough homogenization and was allowed to form a gel through the ionic cross-linking process between sodium alginate molecules [16]. The resulting hydrogel contained RSV at a concentration of 1.2%, encapsulated within the gel matrix composed of sodium alginate (Figure 1). This formulated hydrogel was then utilized in the clinical study.

Periodontal therapy

**Figure 2:**
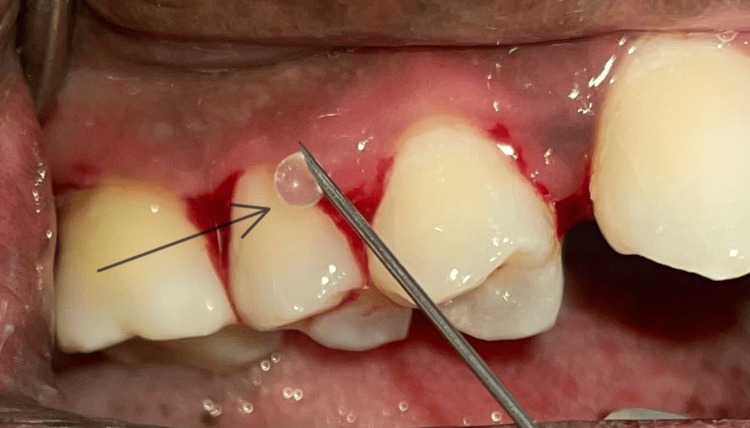
Administration of rosuvastatin hydrogel into the periodontal pocket The arrow indicates RSV hydrogel administration with the syringe.

All participants in this study underwent SRP, which involved the use of both ultrasonic scalers and Gracey curettes (Hu-Friedy®, Chicago, IL). In Group B, the application of 1.2% RSV hydrogel was done along with SRP. The hydrogel was administered subgingivally into the pocket with an invasive syringe (Figure 2). Following the procedure, periodontal dressing (COE-PAK™, GC America Inc., Alsip, IL) was applied to both groups. Participants were instructed to refrain from using interdental aids, consuming hard and sticky foods, and brushing in the operated areas for one week. Following this duration, the periodontal dressing was taken off. Patients were subsequently scheduled for a follow-up examination three months later.

Statistical analysis

Statistical analysis utilized SPSS, version 23.0 software (IBM Corp, Armonk, NY). The Shapiro-Wilk test was employed to evaluate the normality of data distribution. Intergroup comparison was executed through an independent t-test, while intragroup comparison between baseline and three-month was scrutinized employing a paired t-test. Significance was determined by a p-value below 0.05.

## Results

Table 1 illustrates the clinical and demographic features of both study groups. Participants who had undergone SRP only (Group A) presented with an average age of 41.20 ± 2.24 years, mean mSBI of 2.13 ± 0.58, PPD of 7.63 ± 1.66 mm, and CAL of 8.86 ± 1.07 mm. This group consisted of 11 male and nine female participants. Individuals treated with SRP along with 1.2% RSV hydrogel (Group B) presented with an average age of 42.63 ± 2.45 years, mean mSBI of 2.34 ± 0.59, PPD of 7.36 ± 1.12 mm, and CAL of 8.56 ± 1.22 mm. Group B comprised nine males and 11 females. 

**Table 1:** Demographic and clinical characteristics of the study population in both groups mSBI, modified sulcular bleeding index; PPD, probing pocket depth; CAL, clinical attachment level.

Parameter	Group A	Group B	p-Value
Age	41.20 ± 2.24	42.63 ± 2.45	0.73
Gender (male/female)	11/9	9/11	0.43
mSBI	2.13 ± 0.58	2.34 ± 0.59	0.85
PPD	7.63 ± 1.66	7.36 ± 1.12	0.50
CAL	8.86 ± 1.07	8.56 ± 1.22	0.53

Table 2 depicts the comparison between the two groups at baseline and three months. At baseline, there were no significant differences in the average values of all the parameters (mSBI, PPD, and CAL) between the two groups (p > 0.05). However, after three months, a statistically significant difference was noted in all parameters (mSBI, PPD, and CAL), indicating superior outcomes in Group B compared to Group A.

**Table 2:** Comparison of clinical parameters between the two groups using an independent t-test mSBI, modified sulcular bleeding index; PPD, probing pocket depth; CAL, clinical attachment level. *Statistically significant.

Parameters	Timeline	Group A (mean ± SD)	Group B (mean ± SD)	p-Value
mSBI	Baseline	2.13 ± 0.58	2.34 ± 0.59	0.85
	3 months	1.37 ± 0.36	1.01 ± 0.29	0.04*
PPD	Baseline	7.63 ± 1.66	7.36 ± 1.12	0.50
	3 months	7.20 ± 0.70	4.63 ± 0.88	0.01*
CAL	Baseline	8.86 ± 1.07	8.56 ± 1.22	0.53
	3 months	7.56 ± 1.01	5.90 ± 1.24	0.02*

Table 3 presents the comparison between baseline and three-month follow-up within each study group. In Group A, a reduction in mSBI from 2.13 ± 0.58 mm to 1.37 ± 0.36 mm, PPD from 7.63 ± 1.66 mm to 7.20 ± 0.70 mm, and CAL from 8.86 ± 1.07 mm to 7.56 ± 1.01 mm was observed. Significant differences were noted in all parameters (mSBI, PPD, and CAL) with a p-value of 0.00. Similarly, in Group B, mSBI decreased from 2.34 ± 0.59 mm to 1.01 ± 0.29 mm, PPD from 7.36 ± 1.12 mm to 4.63 ± 0.88 mm, and CAL from 8.56 ± 1.22 mm to 5.90 ± 1.24 mm. Significant differences were noted in all values (mSBI, PPD, and CAL) with a p-value of 0.00. 

**Table 3:** Comparison of clinical parameters within each group from baseline to three-month assessment by paired t-test mSBI, modified sulcular bleeding index; PPD, probing pocket depth; CAL, clinical attachment level. *Statistically significant.

Parameters	Timeline	Group A (mean ± SD)	p-Value	Group B (mean ± SD)	p-Value
mSBI	Baseline	2.13 ± 0.58	0.020*	2.34 ± 0.59	0.010*
	3 months	1.37 ± 0.36		1.01 ± 0.29	
PPD	Baseline	7.63 ± 1.66	0.012*	7.36 ± 1.12	0.00*
	3 months	7.20 ± 0.70		4.63 ± 0.88	
CAL	Baseline	8.86 ± 1.07	0.010*	8.56 ± 1.22	0.00*
	3 months	7.56 ± 1.01		5.90 ± 1.24	

## Discussion

Research in the etiology and pathophysiology of periodontal disorders has resulted in the approval of the usage of pharmacological agents. LDD systems deliver the drug to the intended locations, attain a high enough concentration, and last long enough to be beneficial. The present study aimed to assess the efficacy of a 1.2% RSV hydrogel as a supplementary treatment alongside SRP for generalized chronic periodontitis. This is the first study of its kind to utilize RSV in the form of hydrogel as LDD.

Hydrogels are ideal for LDD due to their biocompatibility, tunable porosity, and high water content. They can encapsulate a variety of drugs and release them in a sustained and localized manner, minimizing systemic side effects. Hydrogels also exhibit stimuli-responsive behavior, allowing for on-demand drug release triggered by specific physiological cues. Their injectability and adaptability to different anatomical sites further enhance their utility. Additionally, biodegradable hydrogels ensure gradual drug release and clearance, with non-toxic degradation products. Overall, these properties make hydrogels promising platforms for precise and efficient LDD systems [16].

In the present investigation, the utilization of RSV as an LDD agent alongside SRP demonstrated significant impacts on the management of periodontitis, as evidenced by clinical and biochemical parameters. Notably, RSV adjunctive therapy exhibited notable reductions in mSBI, PPD, and CAL. The unique contribution of this study lies in its pioneering assessment of RSV gel's efficacy as an LDD agent, an aspect that, to our knowledge, has not been thoroughly investigated in previous research. While direct comparisons with other investigations may not be feasible due to the novelty of this approach, the findings of the present study align indirectly with earlier research endeavors. These investigations have explored the adjunctive use of RSV gel and other statins alongside SRP in periodontitis management, demonstrating promising outcomes concerning clinical and microbiological parameters.

Pankaj and colleagues conducted a study comparing the efficacy of locally administered 1.2% RSV gel and 1% metformin (MF) gel as adjuncts to SRP for managing intra-bony defects in patients with chronic periodontitis. Their results demonstrated that the mean reduction in probing depth (PD), CAL gain, and percentage of bone fill were notably higher in the 1.2% RSV gel group compared to the 1% MF gel and placebo group [17]. In another investigation by Shruti Garg et al. in 2017, the effectiveness of 1.2% RSV and 1.2% atorvastatin (ATV) gels as adjunctive treatment to SRP for managing Class II furcation defect was explored. Ninety patients were divided into three groups: SRP with a placebo gel, 1.2% RSV gel, or 1.2% ATV gel. Clinical and radiographic assessments were conducted at baseline, six months, and nine months. The findings revealed that the RSV group exhibited a greater reduction in PD, increased gain in vertical and horizontal CAL, and a significantly higher percentage of defect depth reduction compared to the ATV group at both time points. These results suggest that 1.2% RSV gel may be more effective than 1.2% ATV gel as an adjunctive therapy for managing mandibular Class II furcation defects alongside SRP [18].

Moreover, a recent meta-analysis has underscored the effectiveness of adjunctive statin delivery in reducing PPD, CAL gain, and bone defect fill in chronic periodontitis. This suggests that statins could offer a promising therapeutic avenue for periodontal regeneration [19]. Another meta-analysis evaluated the impact of locally delivered statins, ranking them based on efficacy for treating chronic periodontitis alongside SRP. This study compared ATV, RSV, and simvastatin. The authors proposed that incorporating local statin therapy alongside SRP provides added benefits in periodontitis treatment, even among individuals with diabetes and smokers. Additionally, they indicated that RSV might be most effective in filling intra-bony defects [20]. Furthermore, it has been demonstrated that the local administration of statins (in particular, ATV and RSV) in adjunct to SRP may result in additional significant improvement in terms of CAL gain and PPD reduction compared with SRP alone [21]. Also, Singh et al. [22] revealed that stains are effective in improving periodontal parameters along with anti-inflammatory, osteoconductive, and antimicrobial effects.

Limitations

Overall, the result of the present study underscores the potential of RSV hydrogel as a promising adjunctive therapy for improving the outcomes of conventional periodontal interventions, thereby offering new avenues for improving periodontal health and patient care. Therefore, these findings justify utilizing statins as adjunctive therapy alongside SRP for treating chronic periodontitis. However, further research is warranted to elucidate the precise mechanism underlying RSV's regenerative effects and to explore its efficacy in various periodontal conditions. Moreover, comparative research involving alternative adjunctive treatments with extended follow-up evaluations would offer valuable perspectives on the most effective utilization of RSV hydrogel in periodontal care strategies.

## Conclusions

In conclusion, the study highlights the promising potential of 1.2% RSV hydrogel as a supplementary treatment alongside SRP for managing generalized chronic periodontitis. Through its targeted effect, RSV demonstrates significant improvement in clinical parameters such as mSBI, PPD, and CAL. These findings suggest that adjunctive therapy with RSV hydrogel enhances the efficacy of conventional periodontal interventions, offering a novel approach.
